# London Hospital

**Published:** 1852-07

**Authors:** 


*London Hospital.—Abscess of the Lung following Pneumonia; Death; Autopsy.* (Under the care of Dr. Pereira.)—The formation of abscess in the lung as a consequence of pneumonia is considered a very rare occurrence; but it is probably still more rare to find a circumscribed accumulation of pus in the pulmonary tissue taking on a semi-gangrenous character. Such events seem in some degree to exclude each other; for the vigorous exudation indispensable for hemming in purulent matter can hardly be contemporaneous with sphacelus of the lung. However this may be, it is plain that in constitutions brought to a state of great debility by excesses, the most unusual pathological phenomena will spring up; and these being at all times instructive, we shall just, from the notes of Mr. Whitby, relate the following case:

John A. T——, aged forty, formerly a solicitor, married, and the father of four children, was admitted, January 18, 1852, under the care of Dr. Pereira. The patient is of a dark and sallow complexion, thin, weak, and exhibiting all the signs of a deteriorated constitution; his habits have been very intemperate, the chief beverage he indulged in being spirits. His health had, however, been tolerably good, until about five months before admission, when he was seized with pain in the right side of the chest, increased by a full inspiration; his habitual cough became very troublesome, the expectoration profuse, and of an offensive odor, pretty similar to that which was noticed on his admission. No medical aid was sought until three weeks since, when rigors had set in; which the patient tried to conquer by large potations of gin-and-water. The pain in the right side of the chest was then somewhat relieved by blisters, &c., but he was soon obliged to apply for admission into the hospital.

When first seen, the patient complained of cough and abundant expectoration; his voice was hoarse, the breath very short, the countenance anxious and sallow, the bowels relaxed, but the appetite still good. The tongue was furred and moist, with an unpleasant taste in the mouth; the pulse rapid and small, and the urine abundant, but very ammoniacal.

On examination of the chest, dulness was found over the right subclavicular and mammary regions, the corresponding parts on the left side being resonant: respiratory murmur, indistinct over a great portion of the right side of the chest, both anteriorly and posteriorly, whilst it was puerile on the left. To the right of the central part of the sternum a peculiar tremulous sound is heard, which has much resemblance with the pulsation of a large vessel through a cavity containing fluid. This sound was not, however, constantly noticed, nor could it be excited by any particular position of the body. The patient was aware of this peculiar bruit; he could himself hear it, and compared it to the noise made by water bubbling from the lid of a tea-kettle. The action of the heart was accelerated, but no other irregularity was detected. As to the voice sounds, pectoriloquy was heard at the base of the right scapula. When inquiring about the patient's sensations, it was found that the right side of the chest was painful, and that the cough was extremely troublesome when he lay on his left side, the easiest posture being the slightly raised dorsal decubitus.

Dr. Pereira ordered bark and ammonia to be taken three times a-day. The symptoms continued pretty well unaltered for the next few days, during which the patient took, first chlorate of potash, and subsequently small doses of sulphate of quinine and morphia. The sputa remained of the same nature, the perspirations at night became very profuse, and the relaxation of the bowels could hardly be restrained by logwood and chalk. Thus the patient progressed for about three weeks, the amphoric murmur on respiration being audible over the right side of the chest, and the heart's action being accompanied by a peculiar gurgling and undulatory sound.

From the physical signs, and the nature of the symptoms which had existed previous to the patient's admission, it was assumed that he had suffered from pleuro-pneumonia, which had resulted in abscess of the lung, with adhesion of its walls to the parietes of the chest, besides effusion into the cavity of the pleura. Thus was a tremulous sound produced with each impulse of the heart, resembling the vibration produced by tapping a phial containing air and fluid. Twenty-six days after admission, the patient died suddenly, after expectorating a large quantity of very offensive matter.

*Post-mortem Examination.*—On viewing the chest, before proceeding to the inspection of the thoracic viscera, it was observed that the right side was larger than the left, and presented a rounded appearance, with the filling up of the intercostal spaces. The thorax, when opened, was found completely filled with pus of a very offensive odor. A large abscess was situated in the right lung, the outer wall of the cavity being adherent to the pleura costalis; and running in front of the lung in a transverse direction, was a broad sinus of communication, extending to the middle mediastinum, which region might be considered as the internal boundary of the abscess. The substance of the lung contained a large quantity of mucus, mixed with air and pus, as well as small tubercles and pieces of lymph. The bronchial tubes were filled with matter similar to that contained in the abscess, and the lung was adherent, over its whole surface, to the parietes of the chest. The left organ was perfectly healthy, but the cavity of the pleura contained about five ounces of serum. The heart was pale and of lax fibre, but otherwise in a normal condition.

It is clear that certain changes in the walls of the above described abscess, or a decomposition of its secretion, must have given rise to the fœtor so characteristic of gangrene. The latter did not, however, exist in the manner usually seen, namely, a crumbling down of the pulmonary tissue, and a dark livid color of parts. A circumstance which is finally worthy of record is, that the fœtor had existed from the commencement of the purulent secretion.—*London Lancet.*

